# New Treatment for Exposed Tooth-Pulp

**Published:** 1899-01

**Authors:** 

**Affiliations:** Toledo, Ohio.


### New Treatment for Exposed Tooth-Pulp.

BY DR. CANFIELD, TOLEDO, OHIO.

Read before the Toledo Dental Society.

To our profession, nothing has been held more sacred than the life of the dental pulp.

Some of the noblest teachers in our ranks are noted for their masterful arguments in favor of the conservative treatment of this organ, and their scientific devices for preserving its vitality when exposed to the bacterial influences of the oral cavity; this is the subject of the few notes and experiences in practice, and of a comparatively new material and treatment of which I shall speak later.

Microscopic investigations teach us that the pulp during the period of youth constantly furnishes inorganic matter to the dentine, as well as providing sufficient organic nutriment for the complete development of the tooth, which, at the stage of its eruption, is neither entirely formed nor properly solidified.

In view of the universal acceptance of this, it must be understood that whatever criticism may be presented against the conservation of an exposed pulp, or whatever argument may be advanced in favor of extripating pulps, has no bearing on the pulps in the teeth of the young or up to the age of the adult; and every argument that may be advanced in favor of removing the pulps of adult teeth equally favors the conservation of these organs in the teeth of the young to an age of full maturity.

Between the ages of five and twenty-five the life of the dental pulp should be considered of the greatest importance, and every means should be used to preserve its vitality; it is frequently a matter of chance whether a tooth with a devitalized pulp will be comfortable, or will need to be extracted on account of an alveolar abscess, even where no inflammatory symptoms exist.

The profession is accustomed to regard a pulpless tooth with more or less discoloration with disfavor, which frequently spoils the aesthetic appearance of what otherwise would be a faultless denture. Under such conditions it is easily understood that our best men use every means that art and science afford to preserve the vitality of the pulp, because they fear the probability of a discolored tooth, even if they escape the worse results of an alveolar abscess.

While always keeping this in mind, I have ever been on the alert to find some reliable treatment. While the capping of freshly exposed and healthy pulps, executed under strict antiseptic measures, has prospects of a lasting success, I have been at a loss for satisfactory measures, if confronted with pulpitis.

In attempting the conservative treatment of pulpitis, we have had to make a distinction between a mild and acute form, the latter giving no chance of recovery, while the former case with all professional skill may and probably will die, and for this reason we grasp at anything new that seemingly brings hope in this direction.

Last February a friend of mine wrote me and called my attention to a new preparation called Iodo-Formagen Cement and told of the success he has had with it, and referred me to an article in the January number, 1898, of *Items of Interest*, which I will here read:

"Formaldehyde is derived from methylic alcohol, out of which it is obtained as a colorless, odorous gas through incomplete oxidation. Through the researches of myself, Dr. Lephowski, Krau and others, it is known that formaldehyde has such an effect on an inflamed pulp that the affected tooth can be filled at one sitting. It is used as an aqueous solution of 40 percent. A piece of cotton saturated with this solution, is applied to the inflamed pulp. By this treatment the desired effect is obtained, but the method is faulty in that the formaldehyde solution causes in the pulp, as well as mucous membrane, very severe pain for several hours. Dr. Lepkowski recommends this method only in cases where lack of time demands it.

Hence the question arose, how to obtain the desired result without pain. Many experiments followed, and Lepkowski assures us that weakening and mixing with cocaine is ineffective. After a series of experiments, I found that the required result could be obtained with pure liquefied carbolic acid, which has the property of uniting clear with the formalin in every form.

After long experiments, which I ended in the spring, 1895 and which brought no satisfactory result, I tried and made use of formaldehyde in the treatment of roots. I prepared a very spongy cement, such as is commonly used for the capping of inflamed pulps. After many trials, I found a cement especially fit for the purpose, which contains marble and eugenol. In both fluid and powder, the freshly produced formaldehyde is introduced while the two are kept separate. By mixing this fluid and powder on a glass slab, the putty-like paste called formagen is obtained. This is applied to the exposed pulp, or, if it is still covered, over the softened dentine. The most violent pulpitis ceases in a few minutes, and the parts harden in about five minutes, so that the tooth can be filled with cement or amalgam.

In the course of somewhat over nine months, there have occurred more than a thousand cases of such teeth which have been successfully treated with formagen, so that they were filled permanently at one sitting. It is asked if the teeth so treated have been preserved with pulps dead or alive. This question is answered by the fact that the pulp after four or six weeks' treatment with formagen fully recovers and thenceforward performs its normal functions. In teeth which have been treated with formagen, the fillings as well as the layers of formagen have been removed, and in every case the exposed pulp has responded with normal sensitiveness.

It is daily asked in what manner the formagen acts upon the teeth. To this question there cannot yet be any definite answer; but the thory, so far as it has been determined, is as follows: the pulps covered with formagen are influenced first by the eugenol, and also by the carbolic acid contained in the fluid, which thus deadens the pain. And during the action of the eugenol, the formaldehyde passes gradually out of the formagen and penetrates into the pulp, and changes the same in a small region around the exposed place. The developing gases and fluid from the pulp chamber are absorbed by the porous putty. And from the effect of the formaldehyde the pulp becomes hard and tough. The parts of the pulp not affected, meanwhile act normally; and from this fact, and from the action of the formaldehyde upon the pulp, it becomes, for the first days after application of formagen, inactive, but in a few weeks is restored to its normal functions."

I immediately sent for and procured the formagen cement and commenced its use, and will give you a few cases of experience I have had with it:

CASE 1, February 18th.—Boy, twelve years old, came in with his mother, who said he wished a first tooth extracted. On examination I found the inferior left first permanent molar with large posterior cavity, and informing her it was a permanent tooth, she wished it saved if possible, but without pain, as he had been suffering agony and was very nervous. Placed the dam on the tooth, I cautiously removed the porous layer of dentine which partially exposed the pulp which I found of a deep red color. After washing out the cavity with tepid water and carefully drying the same, I introduced a small portion of formagen cement endeavoring to spread carefully and cover its entire pulp taking care to avoid pressure; the tooth was then filled with Bitter's cement and the patient left the office entirely free from pain; saw tooth in four days, found it all right, with no pain or soreness; saw the tooth in July, found it in good condition and every reason to believe the nerve to be alive.

CASE 2, March 2nd.—Lady, about twenty-five years old, very nervous and fidgety; had been suffering for several days with the tortures of, (well, never mind); could not have tooth out or touched. With much persuasion she took the chair, found superior right bicuspid aching badly with mild pulpitis, I proceeded nearly the same as in former case. After spreading the formagen over the pulp and waiting about five minutes the pain had entirely left, filled the tooth with amalgam; saw the tooth in Jnne, it had given her no trouble and she was happy over the result. I think the nerve is alive to-day.

CASE 3.—Boy, nine years old. Inferior right six-year-old molar, large buccal cavity, tooth soft and chalky, with much pain; cavity extended to posterior root. Proceeded in same manner only with much difficulty, as boy was nervous, mother also, he had been told by all associates that he was going to get killed, but succeeded in my plan, filled tooth with amalgam on top of formagen. This was in June, saw the tooth October 3d, it was all right and had given no trouble.

I do not want to praise the new cement capping too highly, as the time is rather short that I have been using it for a final opinion. But I have a record of about twelve cases, and have yet to know of a failure. Would like you to experiment for yourselves.

